# The association between depression, socio-economic factors and dietary intake in mothers having primary school children living in Rey, south of Tehran, Iran

**DOI:** 10.1186/2251-6581-11-26

**Published:** 2012-12-12

**Authors:** Moloud Payab, Ahmad-reza Dorosty Motlagh, Mohammadreza Eshraghian, Reza Rostami, Fereydoun Siassi, Behnood abbasi, Mehrnaz Ahmadi, Tina Karimi, Mohammad Yoosef Mahjouri, Soroush Seifirad

**Affiliations:** 1 School of Public Health & Institute of Public Health Researches, Tehran University of Medical Sciences, Tehran, Iran; 2 Institute for Psychology and Educational Sciences, University of Tehran, Tehran, Iran; 3 Faculty of Nutrition & Food Technology, Shahid Beheshti University of Medical Science and Health Service, Tehran, Iran; 4 Sciences and Research Branch, Islamic Azad University, Tehran, Iran; 5 Endocrinology and Metabolism Research Center, Tehran University of Medical Sciences, Tehran, Iran

**Keywords:** Depression, Diet, Mothers, Socio-economic factors

## Abstract

**Background:**

According to the WHO report released in 2000, about 121 million people worldwide suffer from depression. The present study aimed to explore factors influencing depression in mothers from Rey, South of Tehran, Iran; who had elementary school children.

**Methods:**

The cross-sectional survey was conducted in spring 2010. Four hundred thirty mothers who had elementary school children, were selected through a two stage cluster sampling. Beck Depression Inventory (BDI) was used to assess depression in the mothers and a 24-hour food recall was used to collect information regarding their dietary intake. General information regarding economic condition and socio-economic status were also gathered using a questionnaire. The data was analyzed using chi-square, one-way analysis of variance and simple regression tests.

**Results:**

In our study, 51.4% of the mothers suffered from depression. There was an inverse correlation between the educational level of the mothers and the heads of household, their occupational status, their marital status, their socio-economic condition and depression. Conversely, any increase in the family size worsened the depression. The daily intake of different macronutrients, except for fat, was lower in individuals of depressed group.

**Conclusion:**

The present study emphasized the fact that more attention should be paid to the educational level and economic condition of the family in order to reduce maternal depression. Family size also plays an important role in this regard.

## Introduction

The major depressive disorder (MDD), also known as unipolar depression, is one of the most common mood disorders experienced during life. The condition is characterized by any changes in appetite, weight, sleeping pattern and routine activities, lack of energy, guilt feeling, difficulty in decision making, lack of motivation and pleasure [1,2].

According to the 2000 WHO report, 121 million people worldwide suffer from depression and MDD is considered as the fourth cause of burden and the most cause of disability worldwide. The report also predicts that MDD will become the second leading cause of burden disease on 2020. It is also mentioned that 15% of patients with MDD eventually commit suicide [3].

In Iran, studies have shown that depression with a prevalence of 3.8% is on the top of the mental disorders and 21% of population suffers from this disease [4]. Hadavi et al. [5] reported that the mild, moderate and major depression prevalence in women referred to Rafsanjan’s health centers respectively was 18%, 19.1% and 3.4%. Also Ahmadi and Yousefi [6] found that in Bakhtiari nomads the overall prevalence of depression is 29.6%. In another study, Kavyani et al. [7] demonstrated that in Tehran, the prevalence of depression in women is 12.6% and in men is 8.47%.

In general, studies have shown that nutritional status and nutrient intake is associated with depression and may be poor diet is a main cause of depression [8].

The prevalence of depression is higher in women; mainly often married women aged 18–24 years who have a child suffering from depression [9]. Considering the fact women constitute half of the population in each society, being depressed reduces their efficacy whether they are working outside or are housekeepers. Regarding this issue, it is clear that depression imposes significant costs on society, therefore studying the association between depression levels and factors, such as nutrients intake in women, could be necessary. To the best of our knowledge, there is no similar study on the association between depression, socio-economic factors and dietary intake in Iran.

Potentially, some of studied factors of this study are preventable, and thus dispelling them could decrease depression rates in the society.

## Methods

### Study population

The cross-sectional study was conducted in spring 2010 on 430 mothers having primary school children living in Rey. In order to obtain the desired number of samples, a pilot study was conducted in Rey elementary schools. The number of regional schools in Rey was identified with the help of “Rey Education Center”. After contacting their administrators, forty three schools were randomly selected among 102 schools. Subsequently, ten students selected from each school and their mothers were invited to participate in the study. Random selection of 430 mothers was commenced with sorting, based on the economic situation, the list of all primary schools (102 public and private schools) in Rey. Thus, the total cumulative frequency of the primary school students was calculated (36039) from the sorted list. Dividing the total cumulative frequency by the number of clusters (43), the distance between clusters (838) was obtained. Selecting a random number from 1–838 and determining their place in the total cumulative frequency, first school was selected. In the second step, in each selected school, in accordance with the chosen grade, about 10 mothers were selected.

Written informed consent was obtained from the patient for publication of this report and any accompanying images.

### Measurements

#### Demographic data

A self report general information questionnaire was used to record the demographic characteristics such as age, marital status, socio-economic characteristics, and education level of all participants.

In order to determine the socio-economic status, participant were asked to specify if household appliances such as furniture, handmade carpet, refrigerator, washing machine, dish washing machine, microwave, personal computer, car, and property are amongst their household appliances. Having 1–3, 4–6, and 7–9 of these nine household appliances, participants were categorized as low, middle, and high socioeconomic status respectively.

#### Body weight and height

Standing height was measured while the subjects had no shoes and their soles were stuck to the wall using a SECA height meter with 0.1 cm accuracy. Weight was measured on using a SECA digital scale with 0.1 kg accuracy, again with subjects having no shoes and minimal clothing. Then Body Mass Index (BMI) was calculated as weight (kg)/height^2^ (m^2^). Weight classification was made based on WHO standard guidelines. In this regard, those with BMI of above 30 were considered as obese, fewer than 18.5 as extremely underweight, between 18.5 and 25 as normal, and between 25 and 30 as overweight [10].

#### Depression assessment

The Persian version of Beck Depression Inventory (BDI) was used to measure depressive symptoms [11]. Individuals gaining higher scores on the 21-question psychological test are more depressed [12]. Thereafter based on their BDI scores, subjects were divided into 6 groups: normal, slightly depressed, required consultation with a psychiatrist, relatively depressed, severely depressed, and excessively depressed (Table 1). Then these groups were combined into three groups: normal, mild to moderate depressed and severely depressed. Many groups were studied the relationship between credibility and reliability of BDI [13]. For instance, internal consistency of BDI 0.73-0.92 with a mean of 0.86 was reported, and the coefficient alpha for psychiatric populations and non-psychiatric populations were measured 0.86 and 0.81 respectively. Beck test-retest reliability with regard to the distance between the two tests and studied population was measured 0.48-0.86 [1,2]. Furthermore, a study on 116 participants in Iran revealed the correlation coefficient of 0.23-0.68 for BDI .

**Table 1:** BDI scores

**Depression status**	**Scores**
Normal	1-10
Slightly depressed	11-16
Require consultation with a psychiatrist	17-20
Relatively depressed	21-30
Severely depressed	31-40
Excessively depressed	>40

#### Dietary assessment methods

The 24 hours Food Recall is a suitable method to evaluate the food intake of different population. The test involves asking subjects to recall and describe all the food and drinks they have consumed in the past 24 hours [14]. In our study the participants completed a 24-hours dietary recall form twice (In the middle of the week, and three days later).

### Statistical analyses

Data were entered and analyzed using Statistical Package for the Social Sciences (SPSS Inc. Chicago, IL, USA). The relationship between qualitative variables with depression levels were assessed using Chi-Square test. The mean and standard deviation were calculated for quantitative variables. Also simple (linear) regression was used to examine the relationship between depression levels and the studied variables. The variables that were significantly associated with depression, were entered into stepwise multiple regression model.

Food intake was converted into nutrients and its grams amount was calculated and encoded. Then, these values were entered in Dorosty Food Processor for Windows software (DFPW- 2.1), and the amounts of energy, carbohydrate, protein and fat intake were calculated.

## Result

### Demographic data

Totally 430 participants were evaluated. The mean age and BMI of the subjects were 34.8 (SD = 5.3) years and 28 (SD = 5) respectively. Also 96.7% of the participants were married and 3.3% were widow. Occupational status of the head of household and socioeconomic, and BMI status respectively in two, fifteen, and one subjects were not recorded. All demographic characteristics are showed in Table 2. The prevalence of depression was 51.4% whereas 46.5% of them suffered from mild to moderate depression, and severe form of the condition was only reported in 4.9%. Depression rate was higher among the older subjects (P = 0.001); it was significantly associated with family size and the number of children (p < 0.001).

**Table 2:** Demographic data (n = 430)

**Characteristics**	**%**
Age*	34.8 **±** 5.3
**Marital status**	
Married	96.7
Widow	3.3
**Occupational status**	
Housekeepers	95.8
Employed	4.2
**Education level**	
Illiterate/Elementary school	16.8
Secondary school/ High school	74.2
University	9
**Occupational status of the head of household (n = 415)**	
Unemployed	3.7
Worker	21.9
Government employee	30
Self-employed	37.2
Retired	3.7
**Education level of the head of household**	
Illiterate/Elementary school	16.1
Secondary school/ High school	65.1
University	18.8
**Residential house ownership status**	
Personal house	55.6
Rent or mortgage	29.1
Living with parents or other relatives	15.3
**Family size**	
Less than 4 persons	30.7
More than 4 persons	69.3
**Socioeconomic status (n = 428)**	
low	32.2
Middle	56.8
High	11

*Data for the age is mean ± SD.

Educational level and occupational status of the mothers and the heads of household, being married and socioeconomic status were inversely associated with depression (Table 3). Based on simple regression fitting, with increasing educational levels of mothers and the heads of household the score of depression were decreased. From among occupations of heads of household, being self-employed was associated with lower rates of depression among the studied population. Also living with of the relatives rather than a personal house, and being widowed was associated with high levels of depression (Table 4).

**Table 3:** Correlation between levels of depression and qualitative variables

**Variables**	**Depression levels**			**Total**	**X**^**2 **^**test / P-Value**
	**Non**	**Mild & Moderate**	**Major**		
**Marital status**					
Married	207(49.8)	192(46.2)	17(4.1)	416(100)	<0.001
Widow	2(14.3)	8(57.1)	4(28.6)	14(100)	
Total	209(48.6)	200(46.5)	21(4.9)		
**Occupational status**					
Housekeepers	199(48.3)	195(47.3)	18(4.4)	412(100)	0.031
Employed	10(55.6)	5(27.8)	3(16.7)	18(100)	
**Education level**					
Illiterate/Elementary school	26(35.6)	41(56.2)	6(8.2)	73(100)	0.025
Secondary school/ High school	158(49.5)	146(45.8)	15(4.7)	319(100)	
University	25(65.8)	13(34.2)	0(0.0)	38(100)	
**Occupational status of the head of household (n = 415)**					
Unemployed	2(12.5)	11(68.8)	3(18.8)	16(100)	0.005
Worker	45(47.9)	45(47.9)	4(4.3)	94(100)	
Government employee	74(57.4)	54(41.9)	1(0.8)	129(100)	
Self-employed	76(47.5)	75(46.9)	9(5.6)	160(100)	
Retired	3(56.3)	7(43.8)	0(0.0)	16(100)	
**Education level of the head of household**					
Illiterate/Elementary school	22(32.8)	39(58.2)	6(9.0)	67(100)	0.013
Secondary school/ High school	143(52.8)	118(43.5)	10(3.7)	271(100)	
University	42(53.8)	35(44.9)	1(1.3)	78(100)	
**Residential house ownership status**					
Personal house	119(49.8)	111(46.4)	9(3.8)	239(100)	0.161
Rent or mortgage	66(52.8)	51(40.8)	8(6.4)	125(100)	
Living with parents or other relatives	24(36.4)	38(57.6)	4(6.1)	66(100)	
**Family size**					
Less than 4 persons	76(57.6)	51(38.6)	5(3.8)	132(100)	0.046
More than 4 persons	133(44.6)	149(50.0)	16(5.4)	298(100)	
**Socioeconomic status (n = 428)**					
low	52(37.7)	74(53.6)	12(8.7)	138(100)	0.003
Middle	127(52.3)	109(44.9)	7(2.9)	243(100)	
High	30(63.8)	15(31.9)	2(4.3)	47(100)	

**Table 4:** Regression fitting between depression and qualitative independent variables

**Variable**	**β ± SE**	**P-value**
**Marital status**		
Constant value	12.34 ± 0.45	<0.001
Being married (Basic group)	-	-
Being widowed	8.73 ± 2.48	0.003
**Occupational status**		
Constant value	12.54 ± 0.46	<0.001
Housekeepers (Basic group)	-	-
Employment	1.96 ± 2.23	<0.719
**Education level**		
Constant value	14.93 ± 1.07	<0.001
Illiterate (Basic group)	-	-
diploma	−2.32 ± 1.19	<0.001
University	−6.59 ± 1.83	<0.001
**Occupational status of the head of household (n = 415)**		
Constant value	19.87 ± 1.61	<0.001
Unemployed (Basic group)	-	-
Worker	−9.19 ± 1.86	0.082
Government employee	−7.3 ± 1.8	<0.001
Self-employed	−9.37 ± 1.77	<0.001
Retired	−6.5 ± 2.77	0.084
**Education level of the head of household**		
Constant value	16.53 ± 1.0	<0.001
Illiterate (Basic group)	-	-
diploma	−4.7 ± 1.15	<0.001
University	−5.22 ± 1.44	<0.001
**Residential house ownership status**		
Constant value	11.91 ± 0.6	<0.001
Personal house (Basic group)	-	-
Rental house	0.88 ± 0.01	0.007
Relatives home	2.97 ± 1.28	0.011

The mean height, weight and BMI had no significant impact on depression. (Tables 5 and 6) The mean daily energy, carbohydrate, and protein intake however had a significant inverse association with depression; such a line was not found for daily fat intake. Moreover, percentage of energy intake from carbohydrates, protein, and fat were not associated with depression ( (Table 7). Based on stepwise multiple regression low socio-economic status, being widowed (In comparison with being married), and having more child were the main factors contributing to depression.

**Table 5:** Correlation between Depression and studied variables

**Variable**	**p-value**	**Pearson correlations**
**Age**	0.014	0.118
**Family size**	> 0.002	0.147
**Number of children**	> 0.001	0.181
**Socio- economic level**	> 0.001	−0.179
**Weight**	0.565	0.028
**Height**	0.224	−0.059
**BMI**	0.277	0.053
**Education level of mother**	0.001	−0.161
**Education level of the head of household**	0.004	−0.142
**Occupational status of the head of mother**	0.022	0.017
**Occupational status of the head of household**	0.002	−0.151
**Residential house ownership status**	0.011	0.077
**Marital Status**	> 0.001	0.182

**Table 6:** Regression fitting between depression and quantitative independent variables

**Variable**	**β ± SE**	**P-value**
**Age**	9.19 ± 2.98	0.002
	0.1 ± 0.08	0.244
**Family size**	6.5 ± 1.91	0.001
	1.53 ± 0.46	0.001
**Number of children**	9.44 ± 1.03	> 0.001
	1.61 ± 0.47	0.001
**Socio- economic Level**	17.79 ± 1.15	> 0.001
	−1.21 ± 0.25	> 0.001
**Weight**	11.42 ± 2.445	>0.001
	0.02 ± 0.03	0.617
**Height**	17.47 ± 12.26	0.155
	−0.03 ± 0.08	0.692
**BMI**	11.02 ± 2.56	> 0.001
	0.06 ± 0.09	0.524

*Constant.

**Coefficient.

**Table 7:** Mothers daily energy and macronutrient intake based on depression levels

**Variables**	**Normal**	**Mild and moderate**	**Major depression**	**Total**	**ANOVA**
	**n = 209**	**depression, n = 200**	**n = 21**	**n = 430**	**P-Value**
**Energy (kcal)**	2246.7 ± 746.8	2110.6 ± 753.3	1698.4 ± 682.9	2158.1 ± 754.5	0.019
**Carbohydrate (gr)**	330.3 ± 120.6	311.4 ± 119.3	248.5 ± 97.8	317.8 ± 120.1	0.033
**Protein (gr)**	64.9 ± 23.7	58.7 ± 19.0	44.5 ± 18.0	61.1 ± 21.8	0.001
**Fat (gr)**	74.0 ± 29.3	70.0 ± 34.7	58.5 ± 31.3	71.4 ± 32.2	0.172
**Percentage of energy intake from carbohydrates**	58.47 ± 6.8	58.66 ± 8.31	59.05 ± 6.03	58.59 ± 7.51	0.478
**Percentage of energy intake from protein**	11.7 ± 2.54	11.49 ± 2.66	10.48 ± 1.51	11.55 ± 2.57	0.11
**percentage of energy intake from fat**	29.83 ± 6.92	29.85 ± 8.49	30.46 ± 6.26	29.87 ± 7.66	0.476

Data are set as the mean ± SD.

## Discussion

The present study reported a high prevalence of depression among Iranian mothers (51.4%). Previous studies have estimated the prevalence of depression in women and men to be 34% and 30% respectively [15]. Kavyani et al. [7] also demonstrated that the prevalence of depression in 20–65 years old women and men living in Tehran was 12.6% and 8.47%. Moreover the prevalence of mild, moderate and major depression in women referred to Rafsanjan’s health centers was 18%, 19.1% and 3.4% respectively [5]. Compared to the other published studies, it seems that depression prevalence in our study is higher than the other studied populations.

Previous studies have highlighted the difference noted in the prevalence of depression in several countries. For example, Ballenger et al. [16,17] reported the prevalence of depression in the United States to have increased from 6% in early 1960 to 28% in early 1990. Furthermore, the results of another study showed that the prevalence of depression among premenopausal and postmenopausal women in Malatya, Turkey was 41.8% [18].

In accordance with several previous studies, our findings indicated a significant positive relationship between depression and the mean age of the participants [5,15,19]. This comes while several studies have shown the contrary [20,21]. On the contrary to the Kerman study, our study revealed a significant positive association between family size and depression [20].

Our findings also showed a significant negative relationship between depression and socio-economic condition. This is consistent with the results of several previous researches [22]. Controversial reports have been stated regarding correlation between income and depression [5,18,21,23]. It seems that inappropriate economic and social conditions can lead to or exacerbate psychological problems in the family members. Furthermore, we found a significant correlation between depression and the occupational status of the head of household; in other words depression was more prevalent among mothers whose husbands were unemployed. Occupational status of the mothers similarly, affected their mood. The condition was more common among housekeepers. Hadavi et al. [5] reported that the prevalence of depression was higher in housekeepers and those married to workers, farmers and self-employed men. Our study however reports a higher rate of depression among women whose husbands were government employee.

In line with many published studies, our findings revealed an inverse correlation between depression and educational levels of the mothers and the heads of household [15,18,19,21]. However, some studies have shown the contrary [19]. It seems that higher educational level can help people adjust better with the environment and have a better performance in dealing with problems and it can improve their mental health.

In addition, our study results revealed a significant correlation between depression and mother’s marital status, so that women who had lost their husbands were more depressed. This association was also reported in several previous published studies [15,18,21,24]. A higher rate of depression among women who have lost their husbands is reported in our study. It was predictable since they are more responsible for their children, additionally lack of support from spouse in the various fields can impose high psychological stress on these women.

The results of present research indicated that the mean daily energy intake significantly and inversely affected depression levels. Moreover, those who took more carbohydrates and protein were significantly less likely to be depressed. Results of Park et al. study [8] contradicts our results in favor of carbohydrate consumption relation with depression while the results of protein consumption in their study were in accordance with our findings.

One of the major limitations of this study is the adopted cross-sectional design. Thus, it was not possible to show the causal relationship between the variables. Moreover, other possible causes of depression were not examined. Further researches are needed to study these variables.

Considering the fact that any change in appetite is a symptom of depression, it is likely the changes in macronutrient intake would lead to depression. It could be concluded that to prevent and reduce the rise of depression, more attention should be paid to education, employment and economic status.

## Competing interests

The authors have no financial competing interests. The data may disclose prevalence of depression in mothers having primary school children living in Rey, South of Tehran, Iran.

## Authors’ contribution

MP participated in the study design, data acquisition, statistical analysis, and interpretation. AD participated in the study design and interpretation. ME participated in the statistical analysis. RR acted as psychology advisor in collection and interpretation of data. FS participated in the study design. BA participated in the data acquisition. MA participated in the data acquisition. TK participated in the data acquisition. MM drafted the manuscript and given final approval of the version to be published. SS drafted the manuscript and submitted the paper, made critical review, and study interpretation, also given final approval of the version to be published.
